# Prevalence, correlates and clinical usefulness of antibodies to RNA polymerase III in systemic sclerosis: a cross-sectional analysis of data from an Australian cohort

**DOI:** 10.1186/ar3544

**Published:** 2011-12-22

**Authors:** Mandana Nikpour, Pravin Hissaria, Jillian Byron, Joanne Sahhar, Maree Micallef, William Paspaliaris, Janet Roddy, Peter Nash, Alan Sturgess, Susanna Proudman, Wendy Stevens

**Affiliations:** 1 The University of Melbourne, Department of Medicine, St. Vincent's Hospital Melbourne, 41 Victoria Parade, Fitzroy, Victoria 3065, Australia; 2 Department of Rheumatology, St. Vincent's Hospital Melbourne, 41 Victoria Parade, Fitzroy, Victoria 3065, Australia; 3 Departments of Clinical Immunology and Immunopathology, Royal Adelaide Hospital, North Terrace, Adelaide, South Australia 5000, Australia; 4 Institute of Medical and Veterinary Science/SA Pathology, 72 King William Road, North Adelaide, South Australia 5000, Australia; 5 Australian Scleroderma Interest Group Project Coordinator, Department of Rheumatology, St. Vincent's Hospital Melbourne, 41 Victoria Parade, Fitzroy, Victoria 3065, Australia; 6 Department of Rheumatology, Monash Medical Centre, 246 Clayton Road, Clayton, Melbourne, Victoria 3168, Australia; 7 Diagnostic Immunology Laboratory, St. Vincent's Hospital Melbourne, 41 Victoria Parade, Fitzroy, Victoria 3065, Australia; 8 Department of Rheumatology, Royal Perth Hospital, Wellington Street (GPO Box X2213), Perth, Western Australia 6001, Australia; 9 Sunshine Coast Rheumatology, PO Box 368, Maroochydore, Sunshine Coast, Queensland 4558, Australia; 10 Department of Rheumatology, The St. George Hospital, Gray Street, Kogarah, New South Wales 2217, Australia; 11 Department of Rheumatology, Royal Adelaide Hospital, North Terrace, Adelaide, South Australia 5000, Australia

## Abstract

**Introduction:**

The prevalence of antibodies to RNA polymerase III (anti-RNAP) differs among systemic sclerosis (SSc) cohorts worldwide. Previously reported associations of anti-RNAP include diffuse cutaneous disease, tendon friction rubs and renal crisis, with recent reports suggesting a close temporal association between malignancy and SSc disease onset among patients with anti-RNAP.

**Methods:**

Patients with SSc were tested for the presence of anti-RNAP at recruitment into the Australian Scleroderma Cohort Study. We used univariate and multivariable methods to identify and quantify clinical and laboratory correlates of anti-RNAP in SSc. Diagnostic testing procedures were used to determine the usefulness of these antibodies in estimating the likelihood of clinically important outcomes.

**Results:**

There were 451 patients with mean ± standard deviation age and disease duration at recruitment of 58.1 ± 12.4 and 11.6 ± 10.0 years, respectively; 151 (33.5%) patients were recruited within 5 years of diagnosis of SSc. Overall, 69 (15.3%) patients had anti-RNAP. Univariate associations of anti-RNAP were diffuse disease (75.4% vs. 20.9%, *P *< 0.0001), joint contractures (73.9% vs. 30.1%, *P *< 0.0001), greater highest-recorded modified Rodnan skin score (20.6 ± 12.4 vs. 10.1 ± 7.9, *P *< 0.0001), synovitis (31.9% vs. 19.9%, *P *= 0.03), myositis (2.9% vs. 0.5%, *P *= 0.05), systemic hypertension (59.4% vs. 39.7%, *P *= 0.002), renal crisis (24.6% vs. 1.8%, *P *< 0.0001) and malignancy diagnosed within 5 years of onset of SSc skin disease (13.3% vs. 3.9%, *P *= 0.01). In multiple regression analysis, after adjustment for other covariates, anti-RNAP were independently associated with renal crisis (odds ratio (OR) 3.8, 95% confidence interval (CI) 1.2 to 11.5, *P *= 0.02; positive predictive value (PPV) 24.6%, negative predictive value (NPV) 98.2%), diffuse disease (OR 6.4, 95% CI 2.9 to 13.8, *P *< 0.0001; PPV 75.4%, NPV 20.9%), joint contractures (OR 2.5, 95% CI 1.2 to 5.3, *P *= 0.02; PPV 73.9%, NPV 69.9%) and malignancy diagnosed within 5 years of onset of SSc skin disease (OR 4.2, 95% CI 1.3 to 13.4, *P *= 0.01; PPV 13.3%, NPV 96.1%).

**Conclusions:**

Anti-RNAP status is a clinically useful prognostic marker in SSc and enables clinicians to identify patients at high risk of developing renal crisis, synovitis, myositis and joint contractures. Patients with anti-RNAP also have an increased risk of malignancy within a 5-year timeframe before or after onset of SSc skin changes.

## Introduction

Systemic sclerosis (SSc) is a multisystem autoimmune disease characterized by vasculopathy and fibrosis [1]. The various manifestations of SSc in affected individuals evolve over time and range from digital ischemia and ulcers to potentially life-threatening renal crisis, interstitial lung disease (ILD) and pulmonary arterial hypertension (PAH). This heterogeneity of clinical manifestations in SSc has led to efforts to find markers that enable identification of patients most at risk of involvement of particular organ systems, who would benefit from more frequent and organ-specific monitoring.

Antibodies to RNA polymerase III (anti-RNAP), detected by immunoprecipitation, were first shown to have specificity for the diagnosis of SSc in the early 1990s [2]. More recently, through the availability of commercial ELISAs, various clinical correlates of anti-RNAP in SSc have been described [3]. Whilst these antibodies are currently thought not to play a pathogenic role, they have prognostic significance. Anti-RNAP appear early in the course of SSc, and, although there is considerable intra-patient and inter-patient variability in antibody titers over time, actual levels do not correlate with disease outcome [4]. A baseline measurement is therefore often sufficient.

The reported frequency of anti-RNAP in various SSc cohorts ranges from 4 to 9.4% in French SSc patients [5-7], to 12% in English SSc patients [8], 6% in Japanese SSc patients [9], 19.4% in Canadian SSc patients [10] and 25% in American SSc patients [7] Racial and genetic variations are hypothesized to account for these differences.

Previously reported associations of anti-RNAP include diffuse cutaneous disease, higher maximum skin thickness score, tendon friction rubs and renal crisis [2,6,8,10-13]. Two recent studies have reported a close temporal association between the onset of SSc and diagnosis of cancer among SSc patients with anti-RNAP [14,15]. This association, however, is yet to be confirmed and quantified in large prospective studies.

In the present study, our objective was to determine the prevalence of anti-RNAP in a large Australian cohort of patients with SSc. We sought to confirm and quantify previously described associations and to define novel clinical and laboratory correlates of these antibodies. We applied diagnostic testing procedures in order to determine the usefulness of these antibodies in estimating the likelihood of clinically important outcomes, including malignancy.

## Materials and methods

### Patients

Consecutive patients recruited into the Australian Scleroderma Cohort Study - a prospective cohort study of risk factors for clinically important outcomes in SSc - were included in the study. These patients fulfilled either the American College of Rheumatology criteria for classification of SSc or the Medsger criteria for limited SSc [16,17]. Patients with mixed connective tissue disease were excluded. Participants were recruited from seven centers specializing in the care of patients with SSc: St. Vincent's Hospital and Monash Medical Centre, Melbourne; Sunshine Coast Rheumatology, Queensland; Royal Adelaide Hospital, South Australia; St George Hospital, Sydney; Royal Perth Hospital, Western Australia; and Prince Charles Hospital, Brisbane. After obtaining informed consent, demographic and disease-related data (including clinical and laboratory variables) were collected, according to a standardized protocol, at recruitment and at each subsequent annual visit. The study was approved by the human research ethics committees of each of the participating centers.

### Measurement of antibodies to RNA polymerase III

Anti-RNAP were measured in serum samples drawn from all patients at recruitment, from 2008 to 2009, using one of two commercial ELISA kits available in Australia: Quanta Lite RNA Pol III (Integrated Sciences, Sydney, NSW, Australia) and MBL Anti-RNA Pol III (Abacus ALS, Brisbane, QLD, Australia). Manufacturer-specified cut-off points were used to define anti-RNAP as present or absent. Both ELISA assays use a purified recombinant immunodominant fragment of RNA polymerase III. Both assays have been validated against an immunoprecipitation assay, with which they have 96% positive agreement and 98% negative agreement. The two ELISA assays are deemed equivalent.

### Demographic, clinical and laboratory variables

Demographic data collected at recruitment included sex, race, age at disease onset and at recruitment into the study. Race was categorized as Caucasian, Asian, Aboriginal or Torres Strait Islander. Disease onset was defined as the date of onset of the first manifestation of SSc, other than Raynaud's phenomenon.

For analysis, data were censored on 31 December 2010. Disease duration at recruitment and also at the time of analysis of data was recorded. Duration of follow-up was defined as the length of time from recruitment to the date at which the data were censored for analysis.

Disease-related data included SSc subtype (limited or diffuse), defined by American College of Rheumatology or Medsger criteria [16,17], and the highest modified Rodnan skin score (mRSS) recorded at the annual follow-up visit [18]. Disease manifestations were defined in each patient as present ever from disease onset to most recent visit. These included Raynaud's phenomenon (with characteristic color changes), digital ulcers and gangrene. Tendon friction rubs, joint contractures and synovitis were diagnosed clinically by the treating physician, at the time of assessment. Myositis was defined as the presence of muscle weakness and/or muscle pain with an elevated serum creatinine kinase, or the presence of inflammatory muscle disease on a muscle biopsy. Creatinine kinase was measured and recorded at each annual visit and the highest level recorded was used for analysis.

Renal crisis was defined as an abrupt onset of severe hypertension (systolic blood pressure (BP) ≥ 180 mmHg and/or diastolic BP ≥ 100 mmHg) without an alternate etiology, with or without microangiopathic anemia or decline in renal function. Serum creatinine, estimated glomerular filtration rate, need for dialysis or renal transplantation, and presence of systemic hypertension (systolic BP ≥ 140 mmHg or diastolic BP ≥ 90 mmHg) were also recorded at each annual visit. Gastrointestinal involvement was defined as one or more of symptomatic or endoscopically proven esophageal reflux, esophageal dysmotility or esophageal stricture, gastric antral vascular ectasia, or symptoms of fecal incontinence or small bowel involvement such as pseudo-obstruction with a positive response to antibiotics, or radiographically proven small bowel involvement either by barium studies or prolonged nuclear transit time. PAH was defined on right heart catheterization as a mean pulmonary artery pressure ≥ 25 mmHg with a pulmonary capillary wedge pressure ≤ 15 mmHg. ILD was defined as the presence of pulmonary fibrosis on high-resolution computerized tomography scanning of the lungs. The need for home oxygen, either for severe PAH or severe ILD, was also recorded. SSc cardiac involvement was defined as the presence of either left ventricular systolic or diastolic dysfunction where no other cause was identified, or a conduction disturbance unexplained by other mechanisms, or a characteristic histological picture on endomyocardial biopsy.

The type and date of diagnosis of malignancies were recorded. We included malignancies that pre-dated or post-dated the diagnosis of SSc. Where applicable, the diagnosis of malignancy required histological confirmation. Malignancies were categorized as solid organ, hematopoietic or skin (nonmelanoma or melanoma). The 'other' category comprised neoplastic variants such as amyloidosis and pre-neoplastic conditions. Smoking was defined as having ever smoked.

SSc treatment was defined in each patient as present ever from disease onset to most recent visit, and was categorized as corticosteroids (including prednisolone but excluding intra-articular injections), antimalarials (hydroxychloroquine), immunosuppressives (methotrexate, azathioprine, mycophenolate mofetil, cyclosporine and cyclophosphamide) and biological therapies (rituximab and TNF antagonists).

At recruitment, in addition to testing for anti-RNAP, all patients also underwent autoantibody profiling using commercially available assays for: antinuclear antibody; antibodies to extractable nuclear antigens (Orgentec ELISA, Mainz, Germany) including antibodies to Scl70, Jo-1, RNP, Ro 60, La, Sm and PM-Scl; antibodies to double-stranded DNA (Amerlex radioimmunoassay; Trinity Biotech, Bray, Ireland); anti-neutrophil cytoplasmic antibodies (Orgentec ELISA) including proteinase-3 or myeloperoxidase specificity; rheumatoid factor; anti-phospholipid antibodies including anti-cardiolipin antibodies (Vital Diagnostics, Bella Vista, NSW, Australia); and anti-β_2 _glycoprotein antibodies (Orgentec ELISA).

### Study design

In a cross-sectional analysis of longitudinally acquired data, demographic, clinical and laboratory correlates (as defined above) of anti-RNAP were determined, using univariate and multiple regression analyses. Among patients ever diagnosed with malignancy, the type and date of diagnosis of cancer relative to the date of SSc disease manifestations - in particular, the date of onset of scleroderma skin changes - was determined.

### Statistical analyses

Univariate methods were used to compare independent variables in patients with and without anti-RNAP. The *t *test was used for continuous variables, and the chi-square test and analysis of variance were used for categorical variables. Logistic and linear regression models were used to identify and quantify independent correlates of anti-RNAP, the highest recorded mRSS, and malignancy diagnosed within 5 years of onset of SSc skin disease. Collinearity between variables was taken into consideration when selecting variables for inclusion in the regression models.

Contingency table analysis was performed to determine the sensitivity, specificity, positive predictive value (PPV) and negative predictive value (NPV) - with corresponding 95% confidence intervals (CIs) - of anti-RNAP for prediction of various SSc disease manifestations in our cohort.

In order to enable unbiased estimation of the association between anti-RNAP and malignancy diagnosed within 5 years of onset of SSc skin disease, we selected patients for these particular analyses as follows: we included all patients diagnosed with malignancy within 5 years - either before *or *after - of onset of SSc skin disease, together with all patients in whom a diagnosis of malignancy had not been made but whose disease duration was at least 5 years at the time of analysis of data.

As there were very few missing data, we did not attempt to impute these data. The number of patients on whom each of the analyses were performed is listed in the results tables.

**Figure 1:**
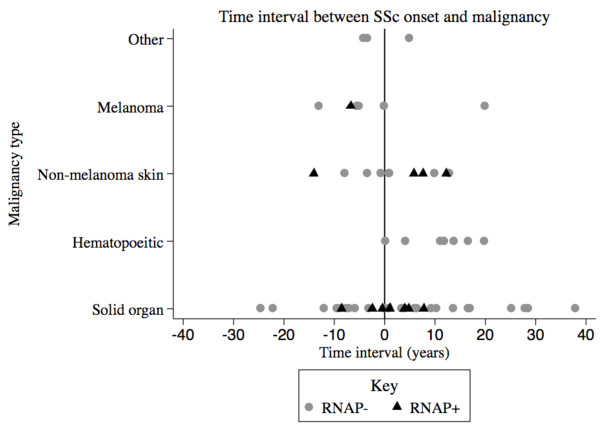
**Time interval between systemic sclerosis onset and diagnosis of malignancy**. Systemic sclerosis (SSc) onset was defined based on onset of skin change. Symbols to the left of the vertical line (denoting time = 0 years) represent malignancies diagnosed prior to the onset of SSc. Solid organ malignancies include breast, lung, colorectal, genitourinary and reproductive tract malignancies. Hematopoeitic malignancies include lymphoma, leukemia and multiple myeloma. Nonmelanoma skin cancers include basal cell and squamous cell carcinomas. The 'other' category includes neoplastic variants and pre-neoplastic disorders. RNAP, anti-RNA polymerase III antibody.

All *P *values were two-tailed and statistical significance was defined as *P *≤ 0.05. All statistical analyses were performed using SAS software version 9.1 (SAS Institute Inc., Cary, NC, USA). Figure 1, a dot plot of the time interval (years) between onset of SSc skin disease and diagnosis of malignancy, according to malignancy type, was generated using STATA software (StataCorp LP, College Station, TX, USA).

## Results

### Patient characteristics

The characteristics of patients in the present study are summarized in Table 1. Among 451 patients who were included in the study, 397 (88.0%) were female, 319 (70.7%) had limited disease, and 132 (29.3%) had diffuse disease. The majority of patients were Caucasian (94.3%), with Asian and Aboriginal-Islander patients comprising 3.9% and 1.8% of the participants, respectively. The mean ± standard deviation (SD) ages at disease onset and at recruitment were 46.5 ± 13.7 and 58.1 ± 12.4 years, respectively. The mean ± SD disease durations at recruitment and at the time of the study were 11.6 ± 10.0 years and 13.6 ± 10.3 years, respectively, in keeping with a group of patients with predominantly prevalent disease. However, 84 (18.6%) patients had been recruited within 2 years of diagnosis, and 151 (33.5%) within 5 years of diagnosis of SSc. From disease diagnosis to the time of the study, all patients manifested Raynaud's phenomenon, while 43% developed digital ulcers, many of them multiple (25% had six or more ulcers during the course of disease). Digital gangrene or amputation occurred in 6.4% of patients. Thirty-two (7.1%) patients had tendon friction rubs and 166 (36.8%) patients had joint contractures. Synovitis and myositis were disease features in 21.7% and 0.9% of patients, respectively. Twenty-four (5.4%) patients developed renal crisis; among these, six (25%) patients required dialysis and two (8.3%) patients had renal transplantation. Systemic hypertension affected 42.8% of patients. All patients had one or more type of gastrointestinal involvement, with 98 (21.7%) patients developing anal incontinence. PAH was diagnosed in 49 (10.9%) patients and ILD in 139 (30.8%) patients. Consequently, nine patients required home oxygen therapy. SSc cardiac involvement occurred in 31 (6.9%) patients. During the mean ± SD follow-up of 1.9 ± 0.8 years, the mean modified Rodnan skin score was 11.7 ± 9.6 points. Overall, 42.0% of patients in the present study had ever smoked.

**Table 1:** Patient characteristics (*n *= 451)

Characteristic	Total^a^	*n *(%) or mean ± SD
Sex	451	
Female		397 (88.0%)
Male		54 (12.0%)
Race	435	
Caucasian		413 (94.3%)
Asian		17 (3.9%)
Aboriginal-Islander		8 (1.8%)
Age (years)		
At disease onset^b^	441	46.5 ± 13.7
At recruitment	448	58.1 ± 12.4
Disease duration (years)		
At recruitment	441	11.6 ± 10.0
At time of the study^c^	441	13.6 ± 10.3
Recruited within 2 years of SSc onset^b^	451	84 (18.6%)
Recruited within 5 years of SSc onset^b^	451	151 (33.5%)
Duration of follow-up^c,d ^(years)	315	1.9 ± 0.8
Disease subtype^e^	451	
Limited		319 (70.7%)
Diffuse		132 (29.3%)
Disease manifestations^f^		
Raynaud's phenomenon	436	436 (100%)
Digital ulcers	451	194 (43.0%)
Tendon friction rub	451	32 (7.1%)
Joint contractures	451	166 (36.8%)
Highest mRSS during follow-up^g^	434	11.7 ± 9.6
Synovitis	451	98 (21.7%)
Myositis	451	4 (0.9%)
Renal crisis^h^	451	24 (5.4%)
Systemic hypertension^i^	444	190 (42.8%)
Gastrointestinal involvement^j^	451	451 (100%)
Pulmonary arterial hypertension^k^	451	49 (10.9%)
Interstitial lung disease^l^	451	139 (30.8%)
Cardiac involvement^m^	451	31 (6.9%)
Serologic profile^n^		
Antinuclear antibodies	451	433 (96.0%)
Anti-centromere pattern	450	197 (43.8%)
Anti-RNA polymerase III antibody	451	69 (15.3%)
Anti-topoisomerase antibodies	446	79 (17.7%)
Anti-histidyl-tRNA synthetase antibodies	446	0 (0%)
Anti-ribonucleoprotein antibodies	445	4 (0.9%)
Anti-Ro antibodies	446	27 (6.1%)
Anti-La antibodies	446	6 (1.4%)
Anti-Smith antibodies	445	1 (0.2%)
Anti-double-stranded DNA antibodies	298	6 (2.0%)
Anti-polymyositis-scleroderma antibodies	447	4 (0.9%)
Anti-neutrophil cytoplasmic antibodies	437	73 (16.7%)
Myeloperoxidase specificity	436	12 (2.8%)
Proteinase-3 specificity	436	15 (3.4%)
Rheumatoid factor	423	126 (29.8%)
Anti-phospholipid antibodies	436	138 (31.7%)
Lupus anticoagulant	325	7 (2.2%)
Treatment^f^	451	
Corticosteroids		198 (43.9%)
Antimalarials^o^		74 (16.4%)
Immunosuppressives^p^		143 (31.7%)
Biological therapies^q^		5 (1.1%)
Smoking ever	436	183 (42.0%)
Malignancy^r^	451	64 (14.2%)
Solid organ^s^		35 (7.8%)
Hematopoietic^t^		7 (1.6%)
Skin (nonmelanoma)^u^		13 (2.9%)
Melanoma		6 (1.3%)
Other^v^		3 (0.7%)
Time interval between SSc onset^w ^and malignancy diagnosis (years)		9.3 ± 8.1

mRSS, modified Rodnan skin score; n (%), number (percentage); SD, standard deviation; SSc, systemic sclerosis. ^a^Total number of patients in whom data were available for analysis. ^b^Disease onset defined as date of onset of first SSc-related manifestation other than Raynaud's phenomenon. ^c^As at 13 December 2010. ^d^Mean duration of follow-up calculated for those who had more than one annual visit. ^e^Disease subtype classified based extent of skin involvement, with limited disease being confined to extremities and face. ^f^Ever from disease onset to most recent visit. ^g^Scores range from 0 to 51, with higher scores indicating more severe skin involvement. ^h^Renal crisis defined as abrupt onset severe hypertension (systolic blood pressure (BP) ≥ 180 mmHg and/or diastolic BP ≥ 100 mmHg) without an alternate etiology, with or without rising creatinine or microangiopathic anemia. ^i^Systolic BP ≥ 140 mmHg and/or diastolic BP ≥ 90 mmHg. ^j^Gastrointestinal involvement defined as one or more of reflux esophagitis, esophageal stricture or dysmotility, gastric antral vascular ectasia, bowel dysmotility or pseudo-obstruction. ^k^Pulmonary arterial hypertension defined as mean pulmonary arterial pressure ≥ 25 mmHg at rest with pulmonary capillary wedge pressure ≤ 15 mmHg. ^l^Interstitial lung disease defined by high-resolution computerized tomography scan of the lung. ^m^Presence of either left ventricular systolic or diastolic dysfunction where no other cause was identified, or a conduction disturbance unexplained by other mechanisms, or a characteristic histological picture on endomyocardial biopsy. ^n^At recruitment. ^o^Hydroxychloroquine. ^p^Includes methotrexate, azathioprine, mycophenolate mofetil, cyclosporine and cyclophosphamide. ^q^Includes TNF antagonists and rituximab. ^r^Ever from birth to most recent visit. ^s^Includes breast, lung, colorectal, genitourinary and reproductive tract malignancies. ^t^Includes lymphoma, leukemia and multiple myeloma. ^u^Includes basal cell and squamous cell carcinomas. ^v^Includes neoplastic variants and pre-neoplastic disorders. ^w^Here SSc onset defined based on onset of skin change.

The autoantibody profile of patients is summarized in Table 1. Overall, 96% had antinuclear antibodies, with 43.8% having a centromere pattern and 17.7% having anti-Scl70 (anti-topoisomerase) antibodies. Sixty-nine (15.3%) patients had anti-RNAP. Other antibodies that were found in a significant proportion of patients were anti-Ro antibodies (6.1%), anti-neutrophil cytoplasmic antibodies (16.7%), rheumatoid factor (29.8%) and anti-phospholipid antibodies (31.7%).

A total of 198 (43.9%) patients had ever been treated with corticosteroids, 74 (16.4%) patients with antimalarials, 143 (31.7%) patients with immunosuppressives and five (1.1%) patients with biological therapies.

### Univariate comparisons

Univariate comparisons of characteristics of patients with (anti-RNAP^+^) and without (anti-RNAP^-^) anti-RNAP are summarized in Table 2. Patients with anti-RNAP were more likely to have diffuse disease (75.4% vs. 20.9%, *P *< 0.0001), joint contractures (73.9% vs. 30.1%, *P *< 0.0001), and greater highest-recorded mRSS (20.6 ± 12.4 vs. 10.1 ± 7.9, *P *< 0.0001), indicating more severe skin involvement. Patients with anti-RNAP were also more likely to have synovitis (31.9% vs. 19.9%, *P *= 0.03) and myositis (2.9% vs. 0.5%, *P *= 0.05). Renal crisis was significantly more prevalent among those with anti-RNAP (24.6% vs. 1.8%, *P *< 0.0001). Among those who developed renal crisis, however, anti-RNAP was not associated with worse outcome as determined by highest serum creatinine, lowest estimated glomerular filtration rate, or the need for dialysis and renal transplantation. Anti-RNAP was also positively associated with systemic hypertension (59.4% vs. 39.7%, *P *= 0.002) and negatively associated with anti-centromere antibodies (5.8% vs. 50.7%, *P *< 0.0001), anti-Scl70 antibodies (1.5% vs. 20.7%, *P *= 0.0001), anti-phospholipid antibodies (13.9% vs. 34.8%, *P *= 0.0008) and rheumatoid factor (12.5% vs. 32.9%, *P *= 0.001), meaning that those with anti-RNAP were less likely to have these other autoantibodies. Those with anti-RNAP were more likely to have been treated with corticosteroids (58.0% vs. 41.4%, *P *= 0.01) and immunosuppressives (58.0% vs. 27.0%, *P *< 0.0001), in keeping with more severe disease.

**Table 2:** Univariate comparison of characteristics of patients with and without antibodies to RNA polymerase III

	Anti-RNAP^+ ^(*n *= 69)		Anti-RNAP^- ^(*n *= 382)		
	
Characteristic	Total^a^	*n *(%) or mean ± SD	Total^a^	*n *(%) or mean ± SD	*P *value
Sex	69		382		
Female		58 (84.1%)		339 (88.7%)	0.27
Male		11 (15.9%)		42 (11.1%)	
Race	65		373		
Caucasian		61 (93.9%)		352 (94.4%)	0.97
Asian		3 (4.6%)		14 (3.8%)	
Aboriginal-Islander		1 (1.5%)		7 (1.9%)	
Age (years)					
At disease onset^b^	68	47.0 ± 11.7	373	46.4 ± 14.0	0.70
At recruitment	68	56.0 ± 12.1	380	58.4 ± 12.4	0.14
Disease duration (years)					
At recruitment	68	9.0 ± 7.9	373	12.1 ± 10.3	0.02
At the time of the study^c^	68	11.4 ± 8.1	373	14.1 ± 10.7	0.06
Duration of follow-up^c ^(years)	59	1.9 ± 0.9	256	1.9 ± 0.8	0.97
Disease subtype^d^	69		382		
Limited		17 (24.6%)		302 (79.1%)	< 0.0001
Diffuse		52 (75.4%)		80 (20.9%)	
Disease manifestations^e^					
Raynaud's phenomenon	66	66 (100%)	370	370 (100%)	1.0
Digital ulcers	69	35 (50.7%)	382	159 (41.6%)	0.16
Tendon friction rubs	69	8 (11.6%)	382	24 (6.3%)	0.12
Joint contractures	69	51 (73.9%)	382	115 (30.1%)	< 0.0001
Highest mRSS during follow-up^f^	68	20.6 ± 12.4	366	10.1 ± 7.9	< 0.0001
Synovitis	69	22 (31.9%)	382	76 (19.9%)	0.03
Myositis	69	2 (2.9%)	382	2 (0.5%)	0.05
Renal crisis^g^	69	17 (24.6%)	382	7 (1.8%)	< 0.0001
Systemic hypertension^h^	69	41 (59.4%)	375	149 (39.7%)	1.0
Gastrointestinal involvement^i^	69	69 (100%)	382	379 (100%)	0.75
Pulmonary arterial hypertension^j^	69	7 (10.1%)	382	42 (11.0%)	0.52
Interstitial lung disease^k^	69	19 (27.5%)	382	120 (31.4%)	0.80
Cardiac involvement^l^	69	7 (10.1%)	382	24 (6.3%)	0.24
Serologic profile^m^	69				
Antinuclear antibodies		67 (97.1%)	382	366 (95.8%)	0.61
Anti-centromere pattern		4 (5.8%)	381	193 (50.7%)	< 0.0001
Anti-topoisomerase antibodies		1 (1.5%)	377	78 (20.7%)	0.0001
Anti-histidyl-tRNA synthetase antibodies		0 (0%)	377	0 (0%)	1.0
Anti-ribonucleoprotein antibodies		1 (1.5%)	376	3 (0.8%)	0.6
Anti-Ro antibodies		3 (4.4%)	377	24 (6.4%)	0.52
Anti-La antibodies		0 (0%)	377	6 (1.6%)	0.29
Anti-Smith antibodies	44	0 (0%)	376	1 (0.3%)	0.67
Anti-double stranded DNA antibodies	69	0 (0%)	254	6 (2.4%)	0.30
Anti-polymyositis-scleroderma antibodies	64	0 (0%)	378	4 (1.1%)	0.39
Anti-neutrophil cytoplasmic antibodies	64	10 (15.6%)	373	63 (16.9%)	0.80
Myeloperoxidase specificity	64	4 (16.3%)	372	8 (2.2%)	0.06
Proteinase-3 specificity	64	2 (13.1%)	372	13 (3.5%)	0.88
Rheumatoid factor	65	8 (12.5%)	359	118 (32.9%)	0.001
Anti-phospholipid antibodies	43	9 (13.9%)	371	129 (34.8%)	0.0008
Lupus anticoagulant	69	0 (0%)	282	7 (12.5%)	0.30
Treatment^e^	69		382		
Corticosteroids		40 (58.0%)		158 (41.4%)	0.01
Antimalarials^n^		10 (14.5%)		64 (16.8%)	0.64
Immunosuppressives^o^		40 (58.0%)		103 (27.0%)	< 0.0001
Biological therapies^p^		1 (1.5%)		4 (1.1%)	0.77
Smoking ever	65	30 (46.2%)	371	153 (41.2%)	0.44
Malignancy^e^	69	14 (13.0%)	382	50 (13.2%)	0.37
Solid organ^q^		9 (13.0%)		26 (6.8%)	
Hematopoietic^r^		0 (0%)		7 (1.8%)	
Skin (nonmelanoma)^s^		4 (5.8%)		9 (2.4%)	
Melanoma		1 (1.5%)		5 (1.3%)	
Other^t^		0 (0%)		3 (0.8%)	
Malignancy diagnosed within 5 years of onset of SSc^u,v^	45	6 (13.3%)	254	10 (3.9%)	0.01
Time interval between SSc onset and malignancy diagnosis (years)^u^	9	4.1 ± 3.1	37	11.7 ± 9.1	0.0002
Malignancy in inception cohort^e,w^	31		120		0.42
Solid organ^q^		4		6	
Hematopoietic^r^		0		1	
Skin (nonmelanoma)^s^		1		3	
Melanoma		0		0	
Other^t^		0		0	

anti-RNAP, anti-RNA polymerase III antibodies; mRSS, modified Rodnan skin score; *n *(%), number (percentage); SD, standard deviation; SSc, systemic sclerosis. ^a^Refers to the total number of patients in whom data were available for analysis. ^b^Disease onset defined as date of onset of first SSc-related manifestation other than Raynaud's phenomenon. ^c^As at 13 December 2010. ^d^Disease subtype classified based extent of skin involvement, with limited disease being confined to extremities and face. ^e^Ever from birth to most recent visit. ^f^Scores range from 0 to 51, with higher scores indicating more severe skin involvement. ^g^Renal crisis defined as abrupt-onset severe hypertension (systolic blood pressure (BP) ≥ 180 mmHg and/or diastolic BP ≥ 100 mmHg) without an alternate etiology, with or without microangiopathic anemia or decline in renal function. ^h^Diastolic BP ≥ 90 mmHg or systolic BP ≥ 140 mmHg. ^i^Gastrointestinal involvement defined as one or more of reflux esophagitis, esophageal stricture or dysmotility, gastric antral vascular ectasia, bowel dysmotility or pseudo-obstruction. ^j^Pulmonary arterial hypertension defined as mean pulmonary arterial pressure ≥ 25 mmHg at rest with pulmonary capillary wedge pressure ≤ 15 mmHg. ^k^Interstitial lung disease defined by high-resolution computerized tomography scan of the chest. ^l^Presence of either left ventricular systolic or diastolic dysfunction where no other cause was identified, or a conduction disturbance unexplained by other mechanisms, or a characteristic histological picture on endomyocardial biopsy. ^m^At recruitment. ^n^Hydroxychloroquine. ^o^Includes methotrexate, azathioprine, mycophenolate mofetil, cyclosporine and cyclophosphamide. ^p^Includes TNF antagonists and rituximab. ^q^Includes breast, lung, colorectal, genitourinary and reproductive tract malignancies. ^r^Includes lymphoma, leukemia and multiple myeloma. ^s^Includes basal cell and squamous cell carcinomas. ^t^Includes neoplastic variants and pre-neoplastic disorders. ^u^Excluding nonmelanoma skin cancers and the 'other' category; here SSc onset defined based on onset of skin change. ^v^Denominator of 299 determined by adding the number of patients who had malignancy diagnosed within 5 years of diagnosis of SSc and all other patients in whom a diagnosis of malignancy had not been made but whose disease duration was ≥ 5 years as of 13 December 2010 (date at which data were censored for analysis). ^w^Patients recruited within 5 years of onset of SSc skin changes.

There were no other statistically significant differences in demographic, disease and treatment related characteristics among anti-RNAP^+ ^and anti-RNAP^- ^patients.

### Regression analyses

In logistic regression modeling, anti-RNAP were independently associated with renal crisis (odds ratio (OR) 3.8, 95% CI 1.2 to 11.5, *P *= 0.02), diffuse disease subtype (OR 6.4, 95% CI 2.9 to 13.8, *P *< 0.0001) and joint contractures (OR 2.5, 95% CI 1.2 to 5.3, *P *= 0.02) (Table 3). In the same model, anti-RNAP was negatively correlated with anti-Scl70 antibodies (OR 0.01, 95% CI 0.002 to 0.11, *P *< 0.0001) and anti-centromere antibodies (OR 0.13, 95% CI 0.04 to 0.41, *P *= 0.0005). After adjustment for these statistically significant covariates, myositis and synovitis were no longer significantly associated with anti-RNAP.

**Table 3:** Independent correlates of antibodies to RNA polymerase III determined using logistic regression

**Variable**^a^	Odds ratio	95% confidence interval	*P *value
Renal crisis^b^	3.8	1.2 to 11.5	0.02
Diffuse subtype^c^	6.4	2.9 to 13.8	< 0.0001
Joint contractures	2.5	1.2 to 5.3	0.02
Anti-topoisomerase antibodies	0.01	0.002 to 0.11	< 0.0001
Anti-centromere antibodies	0.13	0.04 to 0.41	0.0005

In this model, after adjustment for other significant covariates, myositis and synovitis were no longer statistically significant and were therefore removed from model. Univariate odds ratio of RNA polymerase III for renal crisis is 17.4 (95% confidence interval 6.9 to 43.9, *P *< 0.0001). ^a^Ever from disease onset (defined as date of onset of first systemic-sclerosis-related manifestation other than Raynaud's phenomenon) to most recent visit. ^b^Renal crisis defined as abrupt onset severe hypertension (systolic blood pressure ≥ 180 mmHg and/or diastolic blood pressure ≥ 100 mmHg) without an alternate etiology, with or without microangiopathic anemia or decline in renal function. ^c^Disease subtype classified based on extent of skin involvement, with limited disease being confined to extremities and face.

Results of linear regression analysis for independent correlates of highest-recorded mRSS (log_10 _transformed to follow a normal distribution) during follow-up are presented in Table 4. Anti-Scl70 antibodies (parameter estimate 4.37, standard error 0.98, *P *< 0.0001), anti-RNAP (parameter estimate 7.67, standard error 1.09, *P *< 0.0001) and joint contractures (parameter estimate 8.47, standard error 0.81, *P *< 0.0001) were each independently associated with highest-recorded mRSS. As the diffuse disease subtype and anti-Scl70 antibodies were highly correlated (collinear), only the latter was included in the model.

**Table 4:** Independent correlates of highest modified Rodnan skin score recorded during follow-up, determined using linear regression

**Variable**^a^	Parameter estimate	Standard error	*P *value
Anti-topoisomerase antibodies	4.37	0.98	< 0.0001
Anti-RNA polymerase III antibodies	7.67	1.09	< 0.0001
Joint contractures	8.47	0.81	< 0.0001

^a^Ever from disease onset (defined as date of onset of first systemic-sclerosis-related manifestation other than Raynaud's phenomenon) to most recent visit.

### Further renal crisis analyses

In 10 of the 24 cases of renal crisis, the diagnosis was based solely on new-onset severe hypertension (systolic BP ≥ 180 mmHg and/or diastolic BP ≥ 100 mmHg) without an alternate etiology. When analyses were limited to the 14 patients in whom the diagnosis was based on new-onset severe hypertension and one or both of microangiopathic anemia or decline in renal function, nine patients had anti-RNAP (13.0% of total of 69 anti-RNAP^+^) while five patients did not have anti-RNAP (1.31% of total of 382 anti-RNAP^-^) (*P *< 0.0001). Further logistic regression analysis, limited to those with renal crisis based on the latter definition, revealed a univariate OR of renal crisis of 6.8 (95% CI 1.9 to 24.8, *P *= 0.004) in the presence of anti-RNAP.

### Malignancy analyses

Among 451 patients in this study, 64 (14.2%) patients had ever been diagnosed with malignancy, either pre-dating or post-dating the onset of SSc. There were 35 (7.8%) patients with solid organ malignancies, seven (1.6%) patients with hematopoietic malignancies, 13 (2.9%) patients with nonmelanoma skin cancers, six (1.3%) patients with melanoma and three (0.7%) patients with other neoplastic variants and pre-malignant conditions. The mean ± SD time interval between onset of SSc skin changes and diagnosis of malignancy was 9.3 ± 8.1 years.

As seen in Table 2, there was no significant difference in the number or type of malignancies ever diagnosed in patients with or without anti-RNAP. Similarly, in a subgroup analysis of 151 patients recruited within 5 years of SSc skin onset, there was no significant difference in the number or type of malignancies in patients with or without anti-RNAP (Table 2). In the group as a whole, however, the time interval between SSc skin onset and the diagnosis of malignancy was shorter among anti-RNAP^+ ^patients (4.1 ± 3.1 vs. 11.7 ± 9.1 years, *P *= 0.0002), and a higher proportion of malignancies were diagnosed within 5 years of onset of SSc skin changes in the anti-RNAP^+ ^group (13.3% vs. 3.9%, *P *= 0.01). The time interval between SSc skin onset and diagnosis of malignancy in anti-RNAP^+ ^and anti-RNAP^- ^patients is depicted as a dot plot in Figure 1.

The results of logistic regression analysis of correlates of malignancy, diagnosed within 5 years of SSc skin onset, are summarized in Table 5. These correlates were anti-RNAP (OR 4.2, 95% CI 1.3 to 13.4, *P *= 0.01) and older age at SSc skin onset (OR 1.10, 95% CI 1.05 to 1.16, *P *= 0.0002). In this model, after adjustment for anti-RNAP and age at SSc skin onset, there was no statistically significant association between smoking, immunosuppressive use, disease subtype, and malignancy.

**Table 5:** Correlates of malignancy diagnosed within 5 years of systemic sclerosis onset^a^, determined using logistic regression^b^

**Variable**^c^	Odds ratio	95% confidence interval	*P *value
Anti-RNA polymerase III antibodies	4.2	1.3 to 13.4	0.01
Age of onset of systemic sclerosis	1.10	1.05 to 1.16	0.0002
Smoker			NS
Immunosuppressives			NS
Diffuse subtype			NS

NS, not significant. ^a^Before or after the onset of systemic sclerosis skin disease. ^b^Excluding nonmelanoma skin cancers and the 'other' category. ^c^Ever from disease onset to most recent visit.

### Test properties of anti-RNAP

Test properties of anti-RNAP for prediction of various clinical manifestations of SSc are summarized in Table 6. The PPV and NPV were determined based on a prevalence of anti-RNAP in our cohort of 15.3%. The prevalence of each of the clinical manifestations is listed in Table 1. Overall, anti-RNAP had low PPV for all of the manifestations listed in Table 6, except for diffuse SSc subtype and joint contractures where the PPV was 75.4% (95% CI 63.5 to 84.9%) and 73.9% (95% CI 61.9 to 83.8%), respectively. The NPV was high for renal crisis (NPV 98.2%, 95% CI 96.3 to 99.3%), myositis (NPV 99.5%, 95% CI 98.1 to 99.9%) and malignancy within 5 years of onset of SSc skin changes (NPV 96.1%, 95% CI 92.9 to 98.1%).

**Table 6:** Test properties of anti-RNAP for the prediction of various clinical manifestations in systemic sclerosis

**Clinical manifestation**^a^	Sensitivity (95% CI)	Specificity (95% CI)	Positive predictive value (95% CI)	Negative predictive value (95% CI)
Diffuse subtype	14.7% (11.2 to 18.8%)	82.5% (73.4 to 89.5%)	75.4% (63.5 to 84.9%)	20.9% (17.0 to 25.4%)
Joint contracture	30.7% (23.8 to 38.3%)	93.7% (90.2 to 96.2%)	73.9% (61.9 to 83.8%)	69.9% (65.0 to 74.5%)
Synovitis	22.5% (14.6 to 32.0%)	86.7% (82.7 to 90.1%	31.9% (21.2 to 44.2%)	80.1% (75.7 to 84.0%)
Myositis^b^	50.0% (6.8 to 93.2%)	85.0% (81.4 to 88.2%)	2.9% (0.4 to 10.1%)	99.5% (98.1 to 99.9%)
Systemic hypertension	21.6% (16.0 to 28.1%)	89% (84.5 92.6%)	59.4% (46.9 to 71.1%)	60.3% (55.1 to 65.3%)
Renal crisis	70.8% (48.9 to 87.4%)	87.8% (84.3 to 90.8%)	24.6% (15.1 to 36.5%)	98.2% (96.3 to 99.3%)
Malignancy^b ^(all types)	21.9% (12.5 to 34.0%)	85.8% (81.9 to 89.1%)	20.3% (11.6 to 31.7%)	86.9% (83.1 to 90.1%)
Malignancy^b,c ^(certain types)	20.8% (10.5 to 35.0%)	85.8% (81.9 to 89.1%)	15.4% (7.6 to 2.6%)	89.7% (86.2 to 92.6%)
Malignancy within 5 years of SSc onset^c^	37.5% (15.2 to 64.6%)	86.2% (81.7 to 90.0%)	13.3% (5.1 to 16.8%)	96.1% (92.9 to 98.1%)

anti-RNAP, anti-RNA polymerase III antibodies; CI, confidence interval; SSc, systemic sclerosis. ^a^Ever from disease onset to most recent visit; except for malignancy, which was ever from birth to most recent visit. ^b^Chi-square *P *> 0.05 in univariate analysis. ^c^Excluding nonmelanoma skin cancers and the 'other' category.

## Discussion

In this cross-sectional analysis of longitudinally acquired data on a large number (*n *= 451) of patients with SSc, anti-RNAP were present in 15.3%. Anti-RNAP were independently correlated with renal crisis and more severe skin manifestations including diffuse extent of skin involvement, higher skin thickness scores and joint contractures.

The 15.3% prevalence of anti-RNAP in our Australian study is comparable with English (12%) and Canadian (19.4%) studies, possibly reflecting a common racial origin in the majority of Caucasian patients in these cohorts [8,10]. Although we had relatively few Asians and Aboriginal-Islanders, the prevalence of anti-RNAP among these patients was comparable with the group as a whole (3/17 (17.6%) in Asians and 1/8 (12.5%) among Aboriginal-Islanders).

The prevalence of anti-RNAP among Australian SSc patients is markedly greater than French (4 to 9.4%) and Japanese (6%) SSc patients [5-7,19]. While genetic and epigenetic factors may account for these differences, selection bias may be an alternate explanation. Patients with a more severe disease phenotype are more likely to be referred to specialized centers such as ours, thus leading to an overestimation of the frequency of anti-RNAP among SSc patients.

Differences in the prevalence of anti-RNAP in various countries have important implications in terms of the usefulness of such antibodies in clinical practice. While sensitivity and specificity are properties that are inherent to a test, the PPV and NPV are prevalence dependent. Furthermore, the frequency of each of the related clinical manifestations of SSc - for example, renal crisis - may differ among cohorts, again impacting PPV and NPV calculations.

Overall, in our cohort, we have shown that one in four patients with anti-RNAP develops renal crisis, while in absence of this antibody only one in 50 patients develops this complication. The 25% PPV of developing renal crisis with anti-RNAP in our cohort is comparable with that reported in French and US cohorts, wherein the PPV of renal crisis in the presence of anti-RNAP was 20% and 25%, respectively [7]. These findings make a case for home BP monitoring and regular renal function tests in patients with anti-RNAP. The role of prophylactic treatment with angiotensin-converting enzyme inhibitors in these patients is yet to be determined. It is worth noting that a significantly higher proportion of anti-RNAP^+ ^patients had ever been treated with corticosteroids than anti-RNAP^- ^patients (58.0% vs. 41.4%, *P *= 0.01). This is likely to reflect the greater severity of disease in the anti-RNAP^+ ^patients, and the association of these antibodies with synovitis. Corticosteroid use has previously been implicated as a risk factor for the development of SSc renal crisis [20]. In logistic regression modeling in our study, however, anti-RNAP were significantly associated with renal crisis, independently of corticosteroid use, which itself was not associated with renal crisis.

In the present study, we have confirmed that anti-RNAP is a marker for more severe SSc skin disease, with three in four patients positive for the antibody developing joint contractures. As the natural history of skin thickening (scleroderma) in a proportion of patients with SSc is progression early on, and regression after a variable period [21], in this prevalent cohort we may have missed the true peak of the mRSS. Nonetheless, we have shown an association between anti-RNAP and highest-recorded mRSS, independent of anti-Scl70 antibodies. Prospective studies with long-term follow-up of patients recruited from disease onset are required in order to evaluate temporal variations in skin disease, in terms of rate and extent of progression and regression, among those with and without anti-RNAP.

Anti-RNAP are almost mutually exclusive of other autoantibodies. We have here confirmed that anti-RNAP are negatively correlated with anti-centromere and anti-Scl70 antibodies and have also shown that this phenomenon applies to other autoantibodies seen in SSc, such as anti-Ro antibodies, anti-neutrophil cytoplasmic antibodies, rheumatoid factor and anti-phospholipid antibodies. These findings point also to a quasi segregation of associated disease manifestations. For example, among patients in our study only one patient had all of renal crisis, PAH and ILD, while only two patients had both renal crisis and PAH, and only four patients had both renal crisis and ILD.

To date, the association between anti-RNAP and musculoskeletal manifestations of SSc has not been fully characterized. While small numbers of patients limited our ability to demonstrate independent associations in this regard, we did find univariate associations between anti-RNAP and each of synovitis and myositis. While the PPV of anti-RNAP for these manifestations was low, NPVs were high: 80.1% (95% CI 75.7 to 84.0%) for synovitis and 99.5% (95% CI 98.1 to 99.9%) for myositis. This means that the occurrence of SSc myositis is highly unlikely in the absence of anti-RNAP. One should, however, note that myositis and scleroderma are among the characteristic features of mixed connective tissue disease, and patients with mixed connective tissue disease were not included in the present study.

The present study has several limitations. Although one-third of the patients in our study were recruited within 5 years of disease onset, the majority had prevalent disease of more than 5 years' duration at enrolment. As anti-RNAP are associated with a more severe disease phenotype, our findings may be affected by survival bias - wherein those with anti-RNAP-related complications such as renal crisis may have succumbed prior to enrolment, leading to an underestimation of the prevalence of the antibody and the severity of its associations. Another limitation of the study is its cross-sectional design. Although disease manifestations and treatment were defined as present ever from disease onset to the time of analysis, the retrospective nature of some of the data increases the possibility of misclassification. While we used prospectively collected data for many variables such as the mRSS, the prevalent nature of our cohort and the relatively short period of follow-up are methodological limitations.

In analyses of the relationship between anti-RNAP and malignancy, we used the exact date of tissue diagnosis of cancer, relative to the date of onset of SSc. Here, the date of onset of SSc was defined as the date of onset of skin changes, as several SSc patients with malignancy had gastroesophageal reflux recorded as their first non-Raynaud's manifestation and we could not be certain that this nonspecific symptom was truly related to SSc.

Notwithstanding the aforementioned limitations, we have here shown a fourfold increased risk of malignancy within 5 years pre-dating or post-dating the onset of SSc skin disease, among those who are positive for anti-RNAP. This association is independent of disease subtype and age of onset of SSc. After adjustment for other significant covariates, we were unable to demonstrate an association between malignancy and treatment with immunosuppressives among our patients. This may have been due to sample size limitations, and further large-scale prospective studies are needed to evaluate these associations.

Sensitivity/specificity analysis revealed that almost 13% of patients who have anti-RNAP develop cancer within 5 years of SSc skin onset. At present, however, there is no evidence that screening for malignancy in newly diagnosed SSc patients with anti-RNAP leads to improved survival, and a PPV of only 13% would mean many worried-well patients. The NPV of anti-RNAP for the diagnosis of malignancy within 5 years of SSc onset is high (NPV 96.1%, 95% CI 92.9 to 98.1%), offering some reassurance.

The malignancies seen among our anti-RNAP^+ ^patients were predominantly solid organ cancers, with one case of melanoma and fours cases of nonmelanoma skin cancers. We did not document any hematopoietic malignancies among the anti-RNAP^+ ^patients. Overall, the most common cancer among the SSc patients in our study was breast cancer, followed by melanoma, colorectal cancer, lymphoma and lung cancer. It must be noted that the majority of patients in our study were women and that Australia as a whole has one of the highest prevalences of melanoma worldwide [22].

When we defined malignancy as present ever we did not show an increased overall risk of cancer in those with anti-RNAP, compared with those without. Given the observed frequencies of cancer in anti-RNAP^+ ^and anti-RNAP^- ^patients, our study was powered to show a sixfold increased risk of malignancy in patients with anti-RNAP (α = 0.05, β = 0.2). The study was therefore underpowered to demonstrate a weaker but still significant association. There are several other possible explanations. As patients had prevalent disease at recruitment, there may again have been a survival bias. Also, in the process of carcinogenesis, many protective and injurious factors are accrued over time and this may alter the longer-term association between malignancy and anti-RNAP in SSc. Alternatively, it is possible that many SSc patients with anti-RNAP die younger as a result of disease-related complications, before they develop cancer.

## Conclusions

The protean clinical correlates of anti-RNAP in our cohort, wherein the prevalence of such antibodies is 15%, makes anti-RNAP one of the most useful antibodies currently available for prognostication in SSc. Important associations of anti-RNAP include increased risk of renal crisis, systemic hypertension, synovitis, myositis, joint contractures and malignancy within a 5-year timeframe before or after the onset of SSc skin disease. Where possible, these antibodies should be tested in all SSc patients at baseline to guide monitoring and follow-up.

## Abbreviations

anti-RNAP: anti-RNA polymerase III antibody; BP: blood pressure; CI: confidence interval; ELISA: enzyme-linked immunosorbent assay; ILD: interstitial lung disease; mRSS: modified Rodnan skin score; NPV: negative predictive value; OR: odds ratio; PAH: pulmonary arterial hypertension; PPV: positive predictive value; SD: standard deviation; SSc: systemic sclerosis; TNF: tumor necrosis factor.

## Competing interests

The authors declare that they have no competing interests.

## Authors' contributions

MN contributed to the study design, collection and analysis of data, interpretation of results, and preparation of the manuscript. PH contributed to collection of data, interpretation of results and preparation of the manuscript. JB contributed to collection and analysis of data, and preparation of the manuscript. JS contributed to collection of data and preparation of the manuscript. MM contributed to collection of data, interpretation of results, and preparation of the manuscript. WP contributed to collection of data and preparation of the manuscript. JR contributed to collection of data and preparation of the manuscript. PN contributed to collection of data and preparation of the manuscript. AS contributed to collection of data and preparation of the manuscript. SP contributed to the study design, collection of data, interpretation of results, and preparation of the manuscript. WS contributed to the study design, collection of data, interpretation of results, and preparation of the manuscript.
